# Coiled-Coil Domain-Containing Protein 45 Is a Potential Prognostic Biomarker and Is Associated with Immune Cell Enrichment of Hepatocellular Carcinoma

**DOI:** 10.1155/2022/7745315

**Published:** 2022-12-29

**Authors:** Shuqing Liu, Lei Jia, Bing Quan, Guang Rong, Min Li, Ronggen Xie, Yanling Cai, Yuzhen Bi, Siyuan Han

**Affiliations:** ^1^Department of Infectious Diseases, SSL Central Hospital of Dongguan, Dongguan, 523000 Guangdong Province, China; ^2^Department of Organ Transplantation, Affiliated Hospital of Guizhou Medical University, Guiyang, 550000 Guizhou Province, China; ^3^Department of Hepatobiliary Surgery, SSL Central Hospital of Dongguan, Dongguan, 523000 Guangdong Province, China; ^4^Department of Nephrology, SSL Central Hospital of Dongguan, Dongguan, 523000 Guangdong Province, China

## Abstract

**Objective:**

The role of coiled-coil domain-containing protein 45 (CCDC45) in the development of hepatocellular carcinoma (HCC) has not been reported. The present study is aimed at investigating the expression and prognosis of CCDC45 in HCC and its relevance to immune infiltration.

**Methods:**

We conducted CCDC45 expression analysis using The Cancer Genome Atlas (TCGA) tumor database, the Human Protein Atlas (HPA) database, and the Tumor Immunological Evaluation Resource (TIMER). We used the University of Alabama at Birmingham Cancer data analysis Portal (UALCAN) database to show the correlation of CCDC45 with clinical features. We examined the prognostic impact of CCDC45 expression levels on HCC patients with the Kaplan-Meier mapper database. Genes coexpressed with CCDC45 and its regulators were also identified using LinkedOmics. The enriched Gene Ontology (GO) categories and associated signaling pathways were estimated using GO, Kyoto Encyclopedia of Genes and Genomes (KEGG), and Gene Set Enrichment Assay (GSEA) pathway data. Correlations between CCDC45 and cancer immune infiltration was analyzed through the TIMER and an integrated repository portal for Tumor-Immune System Interactions (TISIDB) databases.

**Results:**

The expression of CCDC45 was elevated in HCC tissues compared to adjacent liver tissues, and overexpression of CCDC45 was significantly correlated with tumor stage. Furthermore, HCC patients with CCDC45 overexpression had a shorter overall survival (OS). Functional network analysis indicated that CCDC45 was involved in homologous recombination, spliceosome, and DNA replication. Interestingly, CCDC45 expression was positively correlated with the level of immune cell infiltration.

**Conclusions:**

CCDC45 is associated with prognosis and immune infiltration of HCC and may be a potential therapeutic target for HCC.

## 1. Introduction

Hepatocellular carcinoma (HCC) is the most common primary liver tumor, but the molecular mechanism of HCC development is not well understood [1, 2]. Despite substantial advances in targeted therapies for HCC (e.g., tyrosine kinase inhibitors, such as sorafenib and lenvatinib), previous studies have shown that most patients with HCC developed acquired resistance to targeted agents (e.g., PD-1) in a relatively short period of time [3–5]. HCC is a highly heterogeneous tumor [6]. Interactions with the HCC cell and tumor microenvironment have provided new directions to investigate the molecular mechanisms by which HCC develops and metastasizes; however, the molecular drivers of HCC carcinogenesis remain unclear [7, 8]. In the current management of HCC, there is a lack of reliable early biological markers [9]. Thus, it is essential to identify reliable molecular markers for the early diagnosis and treatment of HCC patients.

Coiled-coil domains constitute a family of proteins containing coiled-coil domains (CCDCs), which are involved in many functions in cell growth and development, such as regulation of gene expression and drug sensitivity [10–15]. The abnormal expression of CCDC65 and CCDC88 proteins has been shown to significantly affect the malignant progression of gastric and pancreatic cancers, providing a theoretical basis for identifying oncology drug targets. Coiled-coil-containing domain 45 (CCDC45) is located on chromosome 17q23.3 of the human genome and encodes a protein of 1200 amino acids. CCDC45 is also known as centrosomal protein 95 (CEP95). Increasing evidence confirms the dysregulated expression of CCDC family genes in tumors, but the expression and clinical significance of CCDC45 in tumors, including HCC, remains uncertain.

In the present study, we utilized several biological databases and multiple experimental approaches to analyze the differential expression of CCDC45 in HCC and its relationship with clinicopathological parameters. Furthermore, we applied Gene Ontology (GO) annotation, Kyoto Gene and Genome Encyclopedia (KEGG), and Gene Set Enrichment Analysis (GSEA) to explore the potential functions of CCDC45 in HCC. In addition, immune infiltration of HCC cells is an important cause of HCC development; hence, we also explored the relevance of immune cells in the immune microenvironment of HCC to the expression of CCDC45. Our findings suggested that CCDC45 may be a potential therapeutic target and a reliable prognostic indicator for HCC.

## 2. Materials and Methods

### 2.1. TCGA Database

Transcriptomic data and clinical information for CCDC45, containing 375 HCC patients and 50 paraneoplastic tissues, were downloaded from The Cancer Genome Atlas (TCGA) database using the UCSC Xena browser, censoring the data cohort used as survival analysis and clinicopathological parameter analysis due to missing data. The transcriptome data were organized into gene expression matrix files using the RSEM program (v1.3.3), and the expression data of CCDC45 in each group of samples were extracted using the R program (v3.6.0). *P* < 0.05 was considered statistically significant.

### 2.2. UALCAN Database

UALCAN is a website that allows online analysis of clinicopathological features of TCGA data, and in this study, we used UALCAN to compare the correlation between CCDC45 and different clinicopathological features, such as tumor grade, stage, patient age, race, and molecular subtype of tumor with gene expression [16]. *P* < 0.05 was considered statistically significant.

### 2.3. Human Protein Atlas (HPA)

The HPA is based on the expression of human proteins detected by proteomics [17]. In the present study, we compared the protein expression of CCDC45 in the liver and HCC tissues from the HPA database.

### 2.4. LinkedOmics Database

The LinkedOmics database is a multiomics site that allows analysis of all tumors in TCGA dataset [18]. In the present study, we queried differentially expressed genes associated with CCDC45 in HCC through the LinkFinder module. Outcomes were analyzed by Pearson correlation coefficients, and these different genes were visualized using volcano plots and heatmaps. To further explore the biological functions that CCDC45 may be involved in, these genes were subjected to GO analysis and KEGG pathway enrichment analysis.

### 2.5. TISIDB Database Analysis

TISIDB is an online analysis of various tumor and immune interactions, aiding in the speculation of the immune effects of immunotherapy [19]. In the present study, the association of CCDC45 expression with immunomodulators and chemokines were analyzed by the TISIDB database. *P* < 0.05 was considered statistically significant.

### 2.6. Tumor Immune Estimation Resource (TIMER) Analysis

TIMER is an online website for the analysis of immune infiltration in different tumors [20, 21]. To investigate the relationship between immunological infiltration of CCDC45 and HCC, we estimated the abundance of immune cells, including B cells, CD8^+^ T cells, CD4^+^ T cells, macrophages, natural killer (NK) cells, and dendritic cells (DCs), through the TIMER algorithm.

### 2.7. GSEA of CCDC45 Expression Phenotype

Based on the expression of CCDC45, all HCC patients were divided into two groups according to high and low expression of CCDC45 using the UCSC Xena platform to download data from TCGA-HCC, and each analysis was repeated 1000 times to align all genes and analyze the enrichment pathways in the CCDC45 high expression group by GSEA [22, 23]. Data were considered statistically significant if *P* < 0.05 and the data were below the FDR-q of 0.25.

### 2.8. RNA Isolation and Quantitative Real-Time Polymerase Chain Reaction (qRT-PCR)

A human normal hepatic epithelial cell line (LO2) and human lung cancer cell lines (HepG2, Hep3B, 97H, and LM3) were purchased from Shanghai Zhongqiao Xinzhou Biotechnology Co., and cultured in Dulbecco's Modified Eagle's Medium (DMEM; Gibco, Grand Island, NY, USA).

Total RNA was routinely extracted after adding TRIzol reagent (Invitrogen, Thermo Fisher Scientific, Shanghai, China) to the cells, and the RNA concentration was determined. cDNA was synthesized and analyzed by qRT-PCR using SYBR® Green (Roche, Basel, Switzerland) [24]. The PCR conditions were 95°C for 10 minutes, 95°C for 15 seconds, and 60°C for 30 seconds for 40 cycles. GAPDH was used as the internal control, and the relative changes between two groups were analyzed by the 2^-*ΔΔ*^Ct method. The following primers were used for qRT-PCR: CCDC45 forward, 5′-AAAAGCCTTAGCCTCACCAAG-3′; CCDC45 reverse, 5′-CTCCCCTAGCTTCCTAGCATT-3′; GAPDH forward, 5′-ATCTTCCAGGAGCGAGATCC-3′; and GAPDH reverse, 3′-ACCACTGACACGTTGGCAGT-5′.

### 2.9. Western Blot Analysis

Cells were lysed sufficiently in protein lysis buffer (radioimmunoprecipitation assay buffer, RIPA) and further fragmented under sonication conditions. After centrifugation, supernatants were collected, and protein levels were determined by a bicinchoninic acid (BCA) protein quantification assay [25]. Proteins were separated on 10% sodium dodecyl-sulfate (SDS) gels and transferred to polyvinylidene fluoride (PVDF) membranes. The membranes were incubated with primary antibodies against CCDC45 (ab230305, Abcam, 1 : 1000) and GAPDH (60004-1-Ig, Ptg, 1: 5000) for 18 hours. After subsequent incubation with secondary antibodies, the luminescence intensity was detected by enhanced chemiluminescence.

### 2.10. Immunohistochemistry

For further determination of CCDC45 expression in HCC, we performed immunohistochemical staining of tissue microarrays from Shanghai Outdo Biotechnology Co. (Shanghai, China). The results of the immunohistochemical staining were double-blinded scored by two pathologists with the rank of Associate Chief Physician. All scores were determined based on staining intensity and range. The intensity of staining was scored based on the following scale: 0, negative expression; 1, weak expression; 2, moderate expression; and 3, strong expression. The staining range was scored based on the following 4-point scale: 0, 0% staining area; 1, staining range of 1% to 24%; 2, staining range of 25% to 49%; 3, staining range of 50% to 75%; and 4, staining range of 75% to 100%. The results of the two separate scores were multiplied to obtain the final score. Patients were included in the low expression CCDC45 group if their total score was <6, while those with a total score ≥ 6 were included in the high expression CCDC45 group.

### 2.11. Survival Analysis

Kaplan-Meier analysis is an online site created by Oncomir to analyze the survival of various genes in relation to tumors. In the present study, we divided CCDC45 into two groups according to their median expression and examined the correlation between CCDC45 in HCC and patient prognosis by log-rank test to determine whether the survival curves of the two groups were statistically different. We also used univariate and multivariate COX regression analyses to validate the relationship between CCDC45 and HCC prognosis. SPSS 22.0 were used to determine significance (*P*) value, and *P* < 0.05 was considered statistically significant.

## 3. Results

### 3.1. CCDC45 Expression Is Increased in HCC

**Figure 1:**
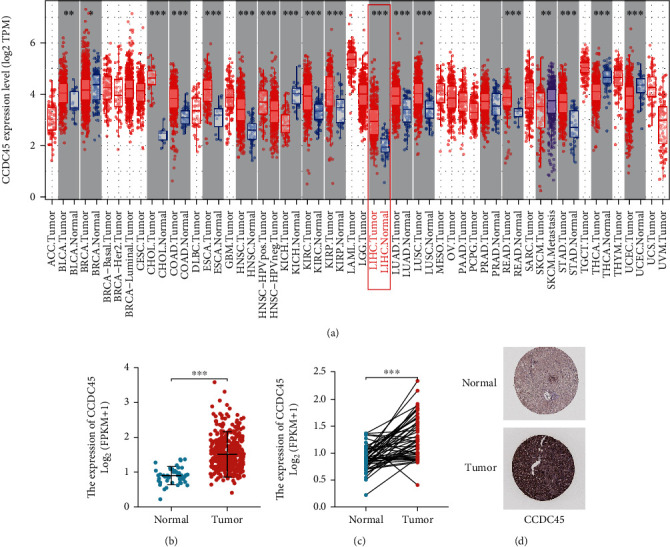
CCDC45 expression levels in HCC patients. (a) Timer2.0 database showing CCDC45 expression levels in different tumors. (b) Expression levels of CCDC45 in unpaired HCC tissues in TCGA database (c) expression levels of CCDC45 in paired HCC tissues in TCGA database. (d) Comparison of CCDC45 expression levels in normal liver tissues and HCC tissues based on the HPA database. ∗*P* < 0.05; ∗∗*P* < 0.01; ∗∗∗*P* < 0.001; ns, not significant, *P* > 0.05.

We used the TIMER2.0 database to explore the expression levels of CCDC45 in normal and various cancer tissues. The results showed that CCDC45 was highly expressed in most tumors, including liver hepatocellular carcinoma (LIHC), and only a few tumors had low CCDC45 expression (Figure 1(a)). A PUBMED database search indicated that the CCDC45 gene has not been reported in tumors. Thus, we focused on CCDC45 in the present study and downloaded TCGA database of HCC-related mRNA expression data to analyze the expression of CCDC45 in HCC. The results demonstrated that CCDC45 was significantly overexpressed in unpaired samples (Figure 1(b)) and paired samples (Figure 1(c)) of HCC, which agreed with the previous pancancer analysis. In addition, CCDC45 protein levels were also higher in HCC tissues than in normal liver tissues in the HPA database (Figure 1(d)). Thus, these findings indicated that CCDC45 expression is upregulated in HCC tissues compared to adjacent liver tissues.

### 3.2. Expression Levels of CCDC45 Correlate with the Clinicopathological Characteristics of HCC Patients

**Figure 2:**
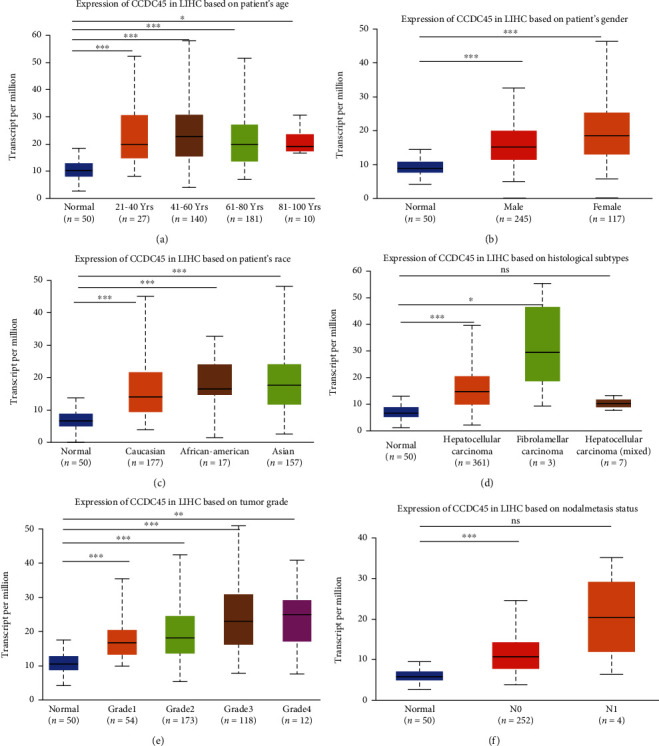
Expression levels of CCDC45 correlate with the clinicopathological characteristics of HCC patients. (a) Age. (b) Gender. (c) Race. (d). Histological subtypes. (e) Tumor grade. (f) Nodal metastasis status.

Subsequently, we examined the expression and clinical characteristics of CCDC45 in HCC using UALCAN. As shown in Figures 2(a)–2(e), CCDC45 correlated with age, gender race, histological type, and tumor stage of HCC patients. These results suggested that increased mRNA expression of CCDC45 may be a diagnostic biomarker in HCC.

### 3.3. Prognostic Potential of CCDC45 in HCC

**Figure 3:**
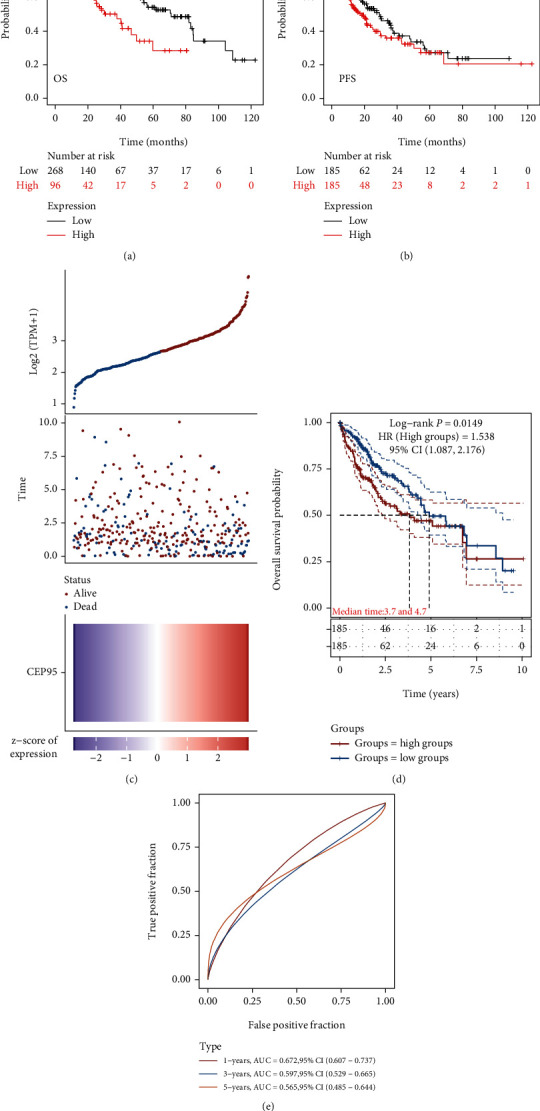
High expression of CCDC45 is associated with poor prognosis in HCC patients. (a) Kaplan-Meier survival analysis of OS of CCDC45 in HCC. (b) Kaplan-Meier survival analysis of PFS of CCDC45 in HCC. (c) Relationship between CCDC45 expression and survival time and survival status in TCGA database. (d) Survival curve of CCDC45 in TCGA database. (e) ROC curves of CCDC45 at different times.

The 10-year overall survival (OS) was better in patients with low CCDC45 expression than in patients with high CCDC45 expression (hazard ratio (HR) = 0.5, 95% confidence interval (CI): 1.31-2.75, *P* = 0.0006, Figure 3(a)). Similar results were obtained for progression-free survival (PFS) (HR = 1.34, 95% CI: 1.00-1.79, *P* = 0.05, Figure 3(b)). Interestingly, CCDC45 expression showed significant differences with the prognosis of HCC patients, and higher CCDC45 expression indicated worse prognosis (Figures 3(c) and 3(d)). The above results suggested that CCDC45 may be a marker that influences disease progression in HCC. Additionally, we further investigated the predictive accuracy and risk score of the effect of CCDC45 on HCC survival using receiver operating characteristic (ROC) curves. CCDC45 was found to be a good predictor of the prognosis of HCC patients at 1, 3, and 5 years with area under the curve (AUC) values of 0.672, 0.597, and 0.565, respectively (Figure 3(e)). Thus, these results indicated that CCDC45 expression is associated with disease progression in HCC patients.

### 3.4. Analysis of CCDC45 Coexpressed Genes in HCC Using GO and KEGG Analyses

**Figure 4:**
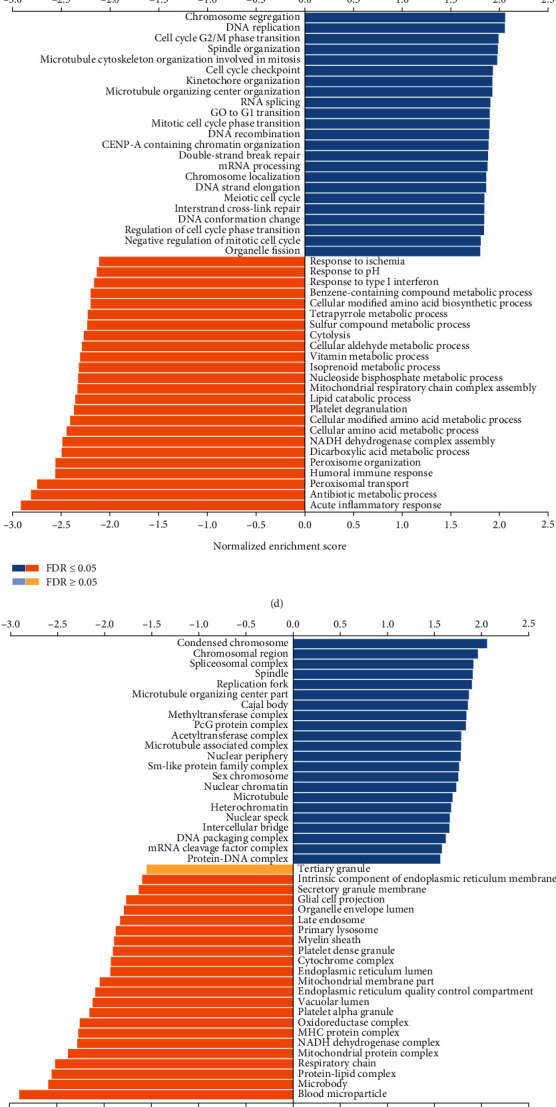
CCDC45 coexpressed genes and GO and KEGG analyses in HCC. (a) Genes coexpressed with CCDC45 in HCC. (b) Top 50 genes positively associated with CCDC45 in HCC. (c) Top 50 genes negatively associated with CCDC45 in HCC. (d) Analysis of GO_BP for CCDC45-associated genes in HCC. (e) Analysis of GO_CC for CCDC45-related genes in HCC. (f) Analysis of GO_MF of CCDC45-associated genes in HCC. (g) KEGG pathway analysis of CCDC45-associated genes in HCC.

To investigate the biological significance of CCDC45 in HCC, we analyzed the coexpression network of CCDC45 in the LIHC cohort through the Linkedomics database. As shown in Figure 4(a) 5,328 genes (red dots) were significantly positively associated with CCDC45, while 2,460 genes (green dots) were significantly negatively associated with ASF1B (false discovery rate, FDR < 0.01). We identified the top 50 genes positively associated with CCDC45 expression and the top 50 genes negatively associated with CCDC45 expression, and they were visualized using a heatmap (Figures 4(b) and 4(c)). The genes most positively associated with CCDC45 included TLK2, DDX42, and POLG2, while those negatively associated with CCDC45 included PINK1, AIFM1, and ACAT1. These genes were subjected to further GO and KEGG enrichment analyses. GO analysis indicated that these genes were enriched in the following GO terms: biological processes, including chromosome segregation, DNA replication, and cell cycle G2/M phase transition (Figure 4(d)); cellular components, including condensed chromosomes, chromosomal regions, spliceosomal complexes, and other related (Figure 4(e)); and molecular functions, including helicase activity, catalytic activity, acting on DNA, and histone binding (Figure 4(f)). KEGG analysis indicated that these coexpressed genes were mainly involved in homologous recombination, spliceosome, and DNA replication (Figure 4(d)). The above results indicated that the coexpression of CCDC45 is closely related to the processes involved in the development of HCC.

### 3.5. Expression of CCDC45 Correlates with the Level of Immune Infiltration in HCC

**Figure 5:**
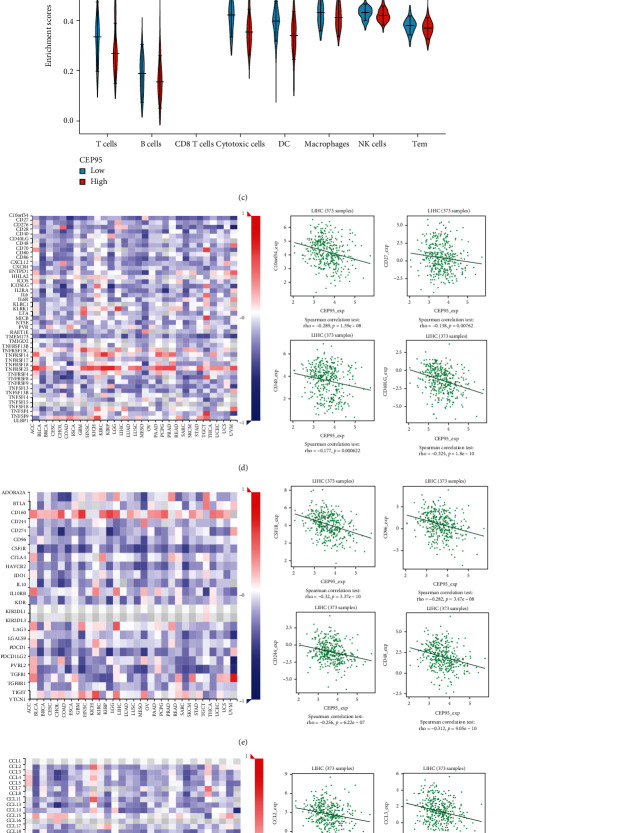
Relationship between CCDC45 expression and immune cell infiltration in HCC. (a) Correlation between CCDC45 expression in the TIMER database and the abundance of tumor-infiltrating immune cells in HCC. (b) Correlation between the expression level of CCDC45 and the degree of immune cell infiltration. (c) Comparison of different levels of immune cell infiltration in the CCDC45 high and low expression groups. (d) Expression of CCDC45 in HCC from the TISIDB database in relation to immunostimulants. (e) Expression of CCDC45 in HCC from the TISIDB database in relation to immunosuppression. (f) Expression of CCDC45 in HCC from the TISIDB database in relation to chemokines. (g) Expression of CCDC45 in HCC from the TISIDB database in relation to chemokine receptors.

To explore the association between the expression level of CCDC45 and immune infiltration, we compared the relationship between the expression of CCDC45 and immune cell cells through the TIMER database. The results demonstrated that CCDC45 was significantly correlated with B cells (Rho = 0.292, *P* = 3.52*e* − 08), CD8+ T cells (Rho = 0.2292, *P* = 3.42*e* − 05), CD4+ T cells (Rho = 0.4, *P* = 1.13*e* − 14), macrophages (Rho = 0.454, *P* = 9.90*e* − 19), NK cells (Rho = 0.373, *P* = 7.89*e* − 13), and DCs (Rho = 0.325, *P* = 8.72*e* − 10) (Figure 5(a)). Intriguingly, high levels of CCDC45 expression significantly correlated with high levels of infiltration of most immune cells, including T cells, helper T cells, NK cells, neutrophils, aDCs, B cells, CD 8^+^ T cells, and regulatory T cells (*P* < 0.05) (Figure 5(b)). The single-sample GSEA (ssGSEA) algorithm was used to determine the correlation between CCDC45 and immune cell enrichment, and the results showed that CCDC45 expression was closely associated with T cells, DCs, NK cells, and macrophages (Figure 5(c)). In addition, CCDC45 was significantly associated with immunostimulants, such as C10orf54 (Rho = −0.289, *P* = 1.59*e* − 08), CD27 (Rho = −0.138, *P* = 008), CD40 (Rho = −0.177, *P* < 0.001), and CD40LG (Rho = −0.324, *P* = 1.80*e* − 10) (Figure 5(b)). The expression of CCDC45 was also associated with immunosuppression, including CSF1R (Rho = −0.320, *P* = 3.37*e* − 10), CD96 (Rho = −0.282, *P* = 3.47*e* − 08), CD244 (Rho = −0.256, *P* = 6.22*e* − 07), and CD48 (Rho = −0.312, *P* = 9.05*e* − 10). CCDC45 expression was also significantly correlated with CCL2 (Rho = -0.241, P = 2.65e-06), CCL3 (Rho = −0.217, *P* = 2.44*e* − 05), CCL4 (Rho = −0.255, *P* = 6024*e* − 07), and CCL5 (Rho = −0.248, *P* = 1.40*e* − 06) (Figure 5(f)). Moreover, CCDC45 expression was significantly associated with chemokine receptors, including CCR1 (Rho = −0.301, *P* = 3.46*e* − 08), CCR2 (Rho = −0.289, *P* = 1.59*e* − 08), CCR4 (Rho = −0.187, *P* < 001), and CCR5 (Rho = −0.280, *P* = 4.21*e* − 08) (Figure 5(g)). These results suggested that CCDC45 may serve as an immunomodulatory factor in HCC.

### 3.6. GSEA of the Signaling Pathways Associated with CCDC45

**Figure 6:**
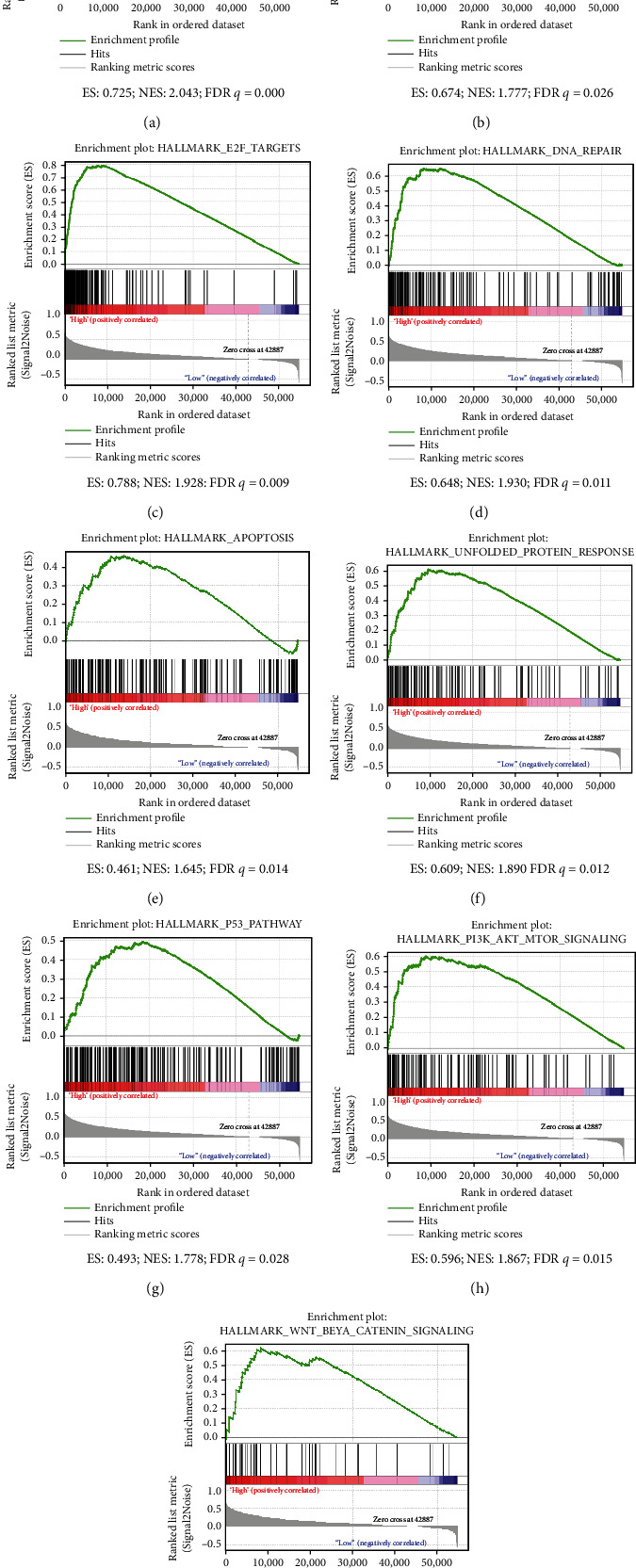
GSEA of the signaling pathways associated with CCDC45. (a) Mitotic spindle. (b) MYC target. (c) E2F target. (d) DNA repair. (e) Apoptosis. (f) Unfolded protein response. (g) P53 pathway. (h) PI3K/AKT/mTOR pathway. (i) Wnt-*β*-catenin pathway.

To further determine the function of CCDC45, GSEA was performed. The results showed that high expression of CCDC45 may be involved with the following pathways: mitotic spindle, MYC target, E2F target, DNA repair, apoptosis, unfolded protein response, P53 pathway, PI3K/AKT/mTOR pathway, and Wnt-*β*-catenin pathway (Figure 6).

### 3.7. CCDC45 Is Highly Expressed in HCC

**Figure 7:**
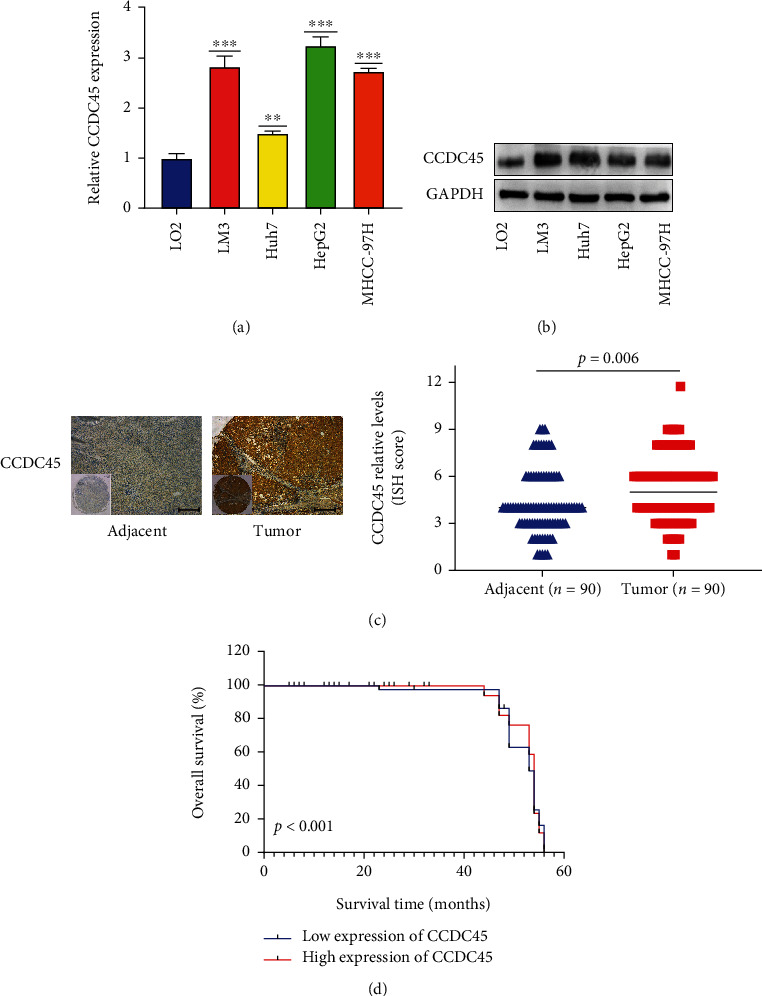
CCDC45 is highly expressed in HCC. (a) Analysis of CCDC45 expression levels in CCDC45 cells by qRT-PCR. (b) Detection of expression levels in CCDC45 cells by Western blot analysis. (c) Detection of CCDC45 expression in clinical tissues by IHC (Scale bar = 50 *μ*m). (d) Kaplan-Meier survival analysis.

For the verification of CCDC45 expression in HCC, we investigated the performance of three HCC cell lines and one immortalized hepatic epithelial cell line (LO2) by qRT-PCR and Western blot analyses to examine the expression level of CCDC45 in cells. Compared to LO2 cells, CCDC45 expression levels were significantly upregulated in HCC cells at both the mRNA (Figure 7(a)) and protein (Figure 7(b)) levels. To further understand the relationship between CCDC45 expression in HCC and clinicopathological parameters of HCC patients, we utilized a tissue microarray containing 90 pairs of HCC tissues and paracancerous tissues for immunohistochemical staining, and the results are shown in Figure 7(c). CCDC45 expression was significantly higher in HCC tissues compared to adjacent paracancerous tissues. Survival analysis showed that high CCDC45 expression was associated with poor prognosis in HCC patients, and patients with low expression of CCDC45 tended to have longer survival times (Figure 7(d)).

We then further assessed the relationship between CCDC45 and the clinicopathological features of HCC by a Chi-square test based on the results of immunohistochemistry (Table 1). CCDC45 expression was associated with Edmondson-Steiner grade (*P* = 0.018). Univariate COX regression analysis showed that American Joint Committee on Cancer (AJCC) stage (*P* = 0.014), GGT (*P* = 0.044), Edmondson-Steiner grade (*P* < 0.001), tumor size (*P* = 0.041), and CCDC45 expression (*P* < 0.001) were associated. Intriguingly, multivariate Cox regression analysis indicated that Edmondson-Steiner grade (*P* = 0.003) and CCDC45 expression (*P* < 0.001) were strongly correlated (Table 2). Thus, these findings suggested that CCDC45 is an independent factor for the prognosis of HCC patients.

## 4. Discussion

With recent advances in precision medicine and immunotherapy, a major breakthrough has been made for the treatment of HCC, but HCC still presents many challenges in the therapeutic field due to its highly heterogeneous nature [26–28]. In addition to molecular pathology research, it is of great interest to discover molecular markers of diagnostic significance in HCC through various bioinformatics tools [8, 29–32].

Investigation of the expression of CCDC family genes in various tumors has been the subject of many studies in recent years [33, 34]. Numerous studies have shown abnormal expression of the CCDC family genes in various tumors, including nonsmall cell lung cancer [35], liver cancer [11], renal cell carcinoma [36], gastric cancer [10], breast cancer [37], and other malignancies. However, CCDC45 has not been reported in HCC. In the present study, we first reviewed databases and found that CCDC45 was upregulated in HCC tissues and that high CCDC45 expression in HCC often predicted a poor prognosis. Moreover, ROC curve analysis suggested that CCDC45 may have predictive value as a biomarker for HCC. Furthermore, we found that CCDC45 overexpression was closely associated with age, gender, race, histological grade, pathological stage, and distant metastasis in HCC patients. The present study is the first to explore CCDC45 expression in HCC by querying multiple bioinformatics databases and combining them with experiments. In the present study, survival analysis indicated that the expression of CCDC45 in HCC was significantly associated with shorter survival, which was supported by several other databases. Multivariate Cox analysis confirmed that CCDC45 is an independent risk factor for HCC patients.

To elucidate the biological role of CCDC45 in HCC, we queried the LinkedOmics database and found that CCDC45 not only had a significant impact on the prognosis of HCC patients but that most of the genes that coexpressed with CCDC45 in HCC also significantly correlated with the prognosis of HCC patients. Furthermore, these coexpressed genes were involved in homologous recombination, spliceosome, and DNA replication. Moreover, GSEA showed that high expression of CCDC45 may be involved in the following pathways: mitotic spindle, MYC target, E2F target, DNA repair, apoptosis, unfolded protein response, P53 pathway, PI3K/AKT/mTOR pathway, and Wnt-*β*-catenin pathway.

HCC cells are involved in a complex tumor microenvironment, including extracellular matrix, stromal cells, and immune cells, which together form the immune microenvironment in which HCC cells grow and metastasize [38–41]. To further examine the relationship and potential mechanisms between CCDC45 and tumor immune microenvironment, we conducted a correlation analysis with immune-related genes. CCDC45 showed positive correlation with the expression of many immune related cells in HCC, including T cells, helper T cells, NK cells, neutrophils, aDCs, B cells, CD8^+^ T cells, and regulatory T cells. Furthermore, poor prognosis of the CCDC45 overexpression group was associated with an overregulation of immune-related suppressor genes and chemokine receptors [42]. Taken together, these findings suggested that targeting CCDC45 may be a potential strategy for immune checkpoint therapy.

Although our multidimensional analysis of CCDC45 and cross-validation using multiple databases provide insight into the association between CCDC45 and HCC to some extent, there were certain limitations in the present study. First, the lack of specificity of the sequencing data due to analyzing different database sources may have introduced sample bias. Moreover, there was a lack of in vitro and in vivo experiments to validate the potential functional effects of CCDC45 on HCC. Thus, future studies focusing on the mechanistic effects of CCDC45 on HCC are required. Although our data suggested that CCDC45 expression is closely associated with immune cell infiltration, the present study lacked direct evidence showing that CCDC45 affects HCC patients through its involvement in immune cell infiltration. Therefore, the mechanism of CCDC45 involvement in the development of HCC through immune regulation remains to be further investigated.

## 5. Conclusions

In summary, the present study demonstrated that CCDC45 is highly expressed in HCC tissues and that high expression of CCDC45 often predicts a poor prognosis. Moreover, the present findings indicated that CCDC45 is an independent prognostic factor for HCC and that the expression of CCDC45 is positively correlated with T cells, B cells, and NK cells. The present study provides new insights into the mechanisms associated with the infiltration of CCDC45 and immune cells in the tumor microenvironment of HCC. We also preliminarily investigated the biological processes associated with CCDC45, providing new directions for further typing studies and clinical applications.

## Figures and Tables

**Table 1:** Correlations between CCDC45 expression and the clinicopathological features of HCC patients.

Characteristics	*n*	CCDC45 expression		*P* value
		Low	High	
Age (years)				
>50	50	25 (50.00%)	25 (50.00%)	1.000
≤50	40	20 (50.00%)	20 (50.00%)	
Gender				
Male	80	39 (48.75%)	41 (51.25%)	0.502
Female	10	6 (60.00%)	4 (40.00%)	
AJCC stage				
I	52	30 (57.69%)	22 (42.31%)	0.088
II-III	38	15 (39.47%)	23 (60.53%)	
HBsAg				
Negative	19	9 (47.37%)	10 (52.63%)	0.796
Positive	71	36 (50.70%)	35 (49.30%)	
AFP (*μ*g/L)				
>400	33	15 (45.45%)	18 (54.55%)	0.512
≤400	57	30 (52.63%)	27 (47.37%)	
Total bilirubin (*μ*mol/L)				
>20	15	8 (53.33%)	7 (46.67%)	0.777
≤20	75	37 (49.33%)	38 (50.67%)	
ALT (U/L)				
>45	31	16 (51.61%)	15 (48.39%)	0.824
≤45	59	29 (49.15%)	30 (50.85%)	
GGT (U/L)				
> 40	59	28 (47.46%)	31 (52.54%)	0.506
≤40	31	17 (54.84%)	14 (45.16%)	
Edmondson-Steiner grade				
I-II	53	32 (60.38%)	21 (39.62%)	**0.018**
III-IV	37	13 (35.14%)	24 (64.86%)	
Tumor number				
Single	79	41 (51.90%)	38 (48.10%)	0.522
Multiple	11	4 (36.36%)	7 (63.64%)	
Tumor size (cm)				
> 5	28	11 (39.29%)	17 (60.71%)	0.172
≤5	62	34 (53.84%)	28 (46.16%)	

**Table 2:** Univariate and multivariate survival analyses of clinicopathological variables of HCC patients.

Characteristics	Overall survival					
	Univariate analysis			Multivariate analysis		
	HR	95% CI	*P* value	HR	95% CI	*P* value
CCDC45 expression	14.635	(4.424-48.412)	**<0.001**	11.413	(3.402-38.285)	**<0.001**
Low vs. high						
Age (years)	1.318	(0.352-1.476)	0.371			
≤50 vs. >50						
Gender	0.536	(0.128-2.249)	0.394			
Male vs. female						
AJCC stage	2.469	(1.197-5.092)	**0.014**	1.655	(0.790-3.466)	0.182
I vs. II-III						
HBsAg	0.692	(0.265-1.806)	0.452			
Negative vs. positive						
AFP (ng/ml)	1.182	(0.574-2.436)	0.650			
≤ 400 vs. >400						
Total bilirubin (*μ*mol/L)	0.930	(0.357-2.424)	0.882			
≤20 vs. >20						
ALT (U/L)	0.867	(0.408-1.812)	0.711			
≤ 45 vs. >45						
GGT (U/L)	0.401	(0.164-0.977)	**0.044**	0.470	(0.184-1.203)	0.115
≤ 40 vs. >40						
Edmondson-Steiner grade	4.985	(2.274-10.928)	**< 0.001**	3.335	(1.497-7.428)	**0.003**
I-II vs. III-IV						
Tumor number	1.887	(0.771-4.621)	0.165			
Single vs. multiple						
Tumor size (cm)	2.103	(1.033-4.281)	**0.041**	1.183	(0.560-2.502)	0.660
≤ 5 vs. >5						

## Data Availability

The bioinformatics data from this study are available in an online database. Additional data are available from the authors upon request.
